# Identification of cuproptosis-related subtypes, construction of a prognosis model, and tumor microenvironment landscape in gastric cancer

**DOI:** 10.3389/fimmu.2022.1056932

**Published:** 2022-11-21

**Authors:** Jinyan Wang, Dongmei Qin, Zhonghua Tao, Biyun Wang, Yizhao Xie, Ye Wang, Bin Li, Jianing Cao, Xiaosu Qiao, Shanliang Zhong, Xichun Hu

**Affiliations:** ^1^ Department of Oncology, Fudan University Shanghai Cancer Center, Shanghai, China; ^2^ Department of Oncology, Shanghai Medical College, Fudan University, Shanghai, China; ^3^ Department of Pathology, Nanjing Jiangning Hospital, The Affiliated Jiangning Hospital of Nanjing Medical University, Nanjing, China; ^4^ Center of Clinical Laboratory Science, Jiangsu Institute of Cancer Research, Jiangsu Cancer Hospital, The Affiliated Cancer Hospital of Nanjing Medical University, Nanjing, China

**Keywords:** cuproptosis-related genes (CRGs), tumor microenvironment (TME), prognosis model, gastric cancer (GC), drugs susceptibility

## Abstract

**Introduction:**

Cuproptosis is a novel identified regulated cell death (RCD), which is correlated with the development, treatment response and prognosis of cancer. However, the potential role of cuproptosis-related genes (CRGs) in the tumor microenvironment (TME) of gastric cancer (GC) remains unknown.

**Methods:**

Transcriptome profiling, somatic mutation, somatic copy number alteration and clinical data of GC samples were downloaded from the Cancer Genome Atlas (TCGA) and the Gene Expression Omnibus (GEO) database to describe the alterations of CRGs from genetic and transcriptional fields. Differential, survival and univariate cox regression analyses of CRGs were carried out to investigate the role of CRGs in GC. Cuproptosis molecular subtypes were identified by using consensus unsupervised clustering analysis based on the expression profiles of CRGs, and further analyzed by GO and KEGG gene set variation analyses (GSVA). Genes in distinct molecular subtypes were also analyzed by GO and KEGG gene enrichment analyses (GSEA). Differentially expressed genes (DEGs) were screened out from distinct molecular subtypes and further analyzed by GO enrichment analysis and univariate cox regression analysis. Consensus clustering analysis of prognostic DEGs was performed to identify genomic subtypes. Next, patients were randomly categorized into the training and testing group at a ratio of 1:1. CRG Risk scoring system was constructed through logistic least absolute shrinkage and selection operator (LASSO) cox regression analysis, univariate and multivariate cox analyses in the training group and validated in the testing and combined groups. Real-time quantitative polymerase chain reaction (RT-qPCR) was used to evaluate the expression of key Risk scoring genes. Sensitivity and specificity of Risk scoring system were examined by using receiver operating characteristic (ROC) curves. pRRophetic package in R was used to investigate the therapeutic effects of drugs in high- and low- risk score group. Finally, the nomogram scoring system was developed to predict patients’ survival through incorporating the clinicopathological features and CRG Risk score.

**Results:**

Most CRGs were up-regulated in tumor tissues and showed a relatively high mutation frequency. Survival and univariate cox regression analysis revealed that LIAS and FDX1 were significantly associated with GC patients’ survival. After consensus unsupervised clustering analysis, GC patients were classified into two cuproptosis molecular subtypes, which were significantly associated with clinical features (gender, age, grade and TNM stage), prognosis, metabolic related pathways and immune cell infiltration in TME of GC. GO enrichment analyses of 84 DEGs, obtained from distinct molecular subtypes, revealed that DEGs primarily enriched in the regulation of metabolism and intracellular/extracellular structure in GC. Univariate cox regression analysis of 84 DEGs further screened out 32 prognostic DEGs. According to the expression profiles of 32 prognostic DEGs, patients were re-classified into two gene subtypes, which were significantly associated with patients’ age, grade, T and N stage, and survival of patients. Nest, the Risk score system was constructed with moderate sensitivity and specificity. A high CRG Risk score, characterized by decreased microsatellite instability-high (MSI-H), tumor mutation burden (TMB) and cancer stem cell (CSC) index, and high stromal and immune score in TME, indicated poor survival. Four of five key Risk scoring genes expression were dysregulated in tumor compared with normal samples. Moreover, CRG Risk score was greatly related with sensitivity of multiple drugs. Finally, we established a highly accurate nomogram for promoting the clinical applicability of the CRG Risk scoring system.

**Discussion:**

Our comprehensive analysis of CRGs in GC demonstrated their potential roles in TME, clinicopathological features, and prognosis. These findings may improve our understanding of CRGs in GC and provide new perceptions for doctors to predict prognosis and develop more effective and personalized therapy strategies.

## Introduction

Gastric cancer (GC) is the fifth most common cancer and the third most common cause of cancer death worldwide (1). Although surgery, chemotherapy, radiotherapy, immunotherapy, and targeted therapy have proven efficacy, the prognosis of GC patients was still poor because of its high recurrence and mortality rate (2). So far, despite increasing studies focused on identifying patients at risk for recurrence and mortality, with the hope of potentially improving outcomes, there were few satisfactory biomarkers or methods that could accurately predict the survival of GC patients (3). Thus, there is an urgent need to identify the prognostic signature and potential mechanism of the development of GC.

Copper is a fundamental trace element involved in a variety of biological processes, including mitochondrial respiration, iron uptake, kinase signaling, autophagy, protein quality control and antioxidant/detoxification processes (4). Increasing pieces of evidence demonstrated dysregulation of copper homeostasis may trigger cytotoxicity and influence tumor growth and metastasis (5). Consistently, serum or tissue levels of copper were elevated in various human cancers, such as breast, brain, prostate, colon, lung and liver cancer (6–13). At the same time, copper chelation and ionophore, such as tetrathiomolybdate (TTM) and disulfiram, have been applied in anticancer treatment (14–16). However, the specific underlying mechanisms by which copper overload leaded to cell death remained unknown, until Tsvetkov et al. (17) discovered that copper toxicity occurred *via* a mechanism different from all other known mechanisms of regulated cell death (RCD), and termed it as cuproptosis. This research demonstrated that cuproptosis occurred by directly binding copper to lipoylated components of the tricarboxylic acid (TCA) cycle, thus leading to the abnormal aggregation of lipoylated protein and loss of iron-sulfur cluster, which ultimately resulted in proteotoxic stress response-mediated cell death. Furthermore, this study identified ten genes (FDX1, LIAS, LIPT1, DLD, DLAT, PDHA1, PDHB, MTF1, GLS, and CDKN2A) closely associated with cuproptosis. Based on the research of Tsvetkov et al., increasing studies are emerging to explore the associations between cuproptosis-related genes (CRGs) and typical tumors. For example, Bian, Z. et al. (18) identified a CRGs signature (FDX1, DLAT and CDKN2A) in clear cell renal cell carcinoma (ccRCC) and found it could serve as a potential prognostic predictor for ccRCC. Chen, Y (19) also identified a cuproptosis-related prognostic signature (CDKN2A, GLS, and LIPT1) for uterine corpus endometrial carcinoma (UCEC). In addition, different cuproptosis-related risk score systems were established respectively based on the comprehensive analysis of CRGs in hepatocellular carcinoma (HCC) and esophageal carcinoma (ESCA) (20, 21). A high cuproptosis-related risk score indicated poor survival, and was positively associated with pro-tumor immune infiltrates in tumor microenvironment (TME) of HCC and ESCA. TME has been recognized as an essential role in regulating tumor immune suppression, distant metastasis, local resistance and the targeted therapy response (22, 23). Specific alterations in TME, such as T-cell exhaustion and activation of epithelial NOTCH signaling, were closely associated with the prognosis of cancer patients (24, 25). The characterization of TME was proposed to predict patients’ survival, chemotherapy and immunotherapy response (26, 27).

However, due to heterogeneity of tumors and corresponding TME, CRGs signature varied in different cancers. Researches on CRGs in GC are limited. Specifically, there is uncertainty regarding the prognostic accuracy of CRGs and their relationships with TME in GC. In this study, we aimed to comprehensive analysis the molecular alterations and clinical relevance of CRGs, through constructing two cuproptosis patterns. We are the first to establish CRGs Risk scoring system to predict GC patients’ survival. This scoring system can provide new perceptions for doctors to develop more effective and personalized therapy strategies.

## Methods

### Data acquisition

We downloaded RNA-Sequence data and the corresponding clinicopathological data of STAD project for stomach adenocarcinoma from The Cancer Genome Atlas (TCGA) (https://portal.gdc.cancer.gov/), and expression levels in TPM of genes were extracted and combined for all samples. Then, series matrix file of GSE84433 was downloaded from the Gene Expression Omnibus database (GEO) (https://www.ncbi.nlm.nih.gov/geo/). The TCGA cohort contained 375 GC samples and 32 normal samples from 375 patients, and the GEO cohort (GSE84433) contained 357 GC samples. The detailed clinicopathological information on these GC patients is presented in **Table S1**. The GEO dataset was combined with TCGA-STAD dataset. Before conducting subsequent analyses, we eliminated batch effects by using “Combat” algorithm. Additionally, somatic mutation data were downloaded from TCGA and contained 431 GC samples. Somatic copy number alteration data were downloaded from TCGA and contained 440 GC samples.

### Consensus clustering analysis of CRGs and differentially expressed genes (DEGs)

Nineteen CRGs, listed in **Table S2**, were achieved from previous cuproptosis-related publications (17, 28). Consensus unsupervised clustering analysis was carried out by using “ConsensusClusterPlus” package in R to classify GC patients into different molecular subtypes according to the expression of CRGs. Furthermore, DEGs, derived from different molecular subtypes, were grouped into different genomic subtypes by the same way. The criteria were as follows: First, the number of samples in each group was relatively consistent. Second, the cumulative distribution function (CDF) curve rose gradually and smoothly. Third, after clustering, the intra-group link was stronger, while the inter-group link was weaker.

### Correlation of molecular subtypes, clinicopathological characteristics and prognosis

To explore the clinical value of different molecular subtypes, we performed correlation analysis between molecular subtypes and clinicopathological characteristics of GC patients. The clinicopathological characteristics included age, gender, grade and tumor node metastasis (TNM) stage. Survival and survminer packages in R were used for survival analysis, the same as our previous research (29). We used Kaplan–Meier plot and log-rank to test the correlations between molecular subtypes and overall survival (OS) of GC patients.

### Relationships between distinct molecular subtypes and TME

In order to verify the characteristics of TME in distinct molecular subtypes, we conducted gene set variation analysis (GSVA) with the hallmark gene set (C2.CP.KEGG (186 gene sets) and C5.GO.Gene Ontology (10561 gene sets)), achieved from the MSigDB database (https://www.gsea-msigdb.org/gsea/msigdb). Adjusted P-value <0.05 was recognized to be statistically significant. Gene Set Enrichment Analysis (GSEA) (4.1.0) based on different gene sets, was applied to learn the specific functional profile of different molecular subtypes. The absolute value of normalize enrichment score (NES) >1, nominal p value<0.05, FDR<0.25 were considered to be statistically significant. In addition, the deconvolution algorithm (referred to as CIBERSORT) was used to calculate the abundance of tumor-infiltrating immune cells (TICs) in each GC sample (30). The gene expression signature matrix of TICs was downloaded from the CIBERSORT platform (https://cibersortx.stanford.edu/). The matrix data of gene expression levels in TCGA-STAD and GSE84433 cohorts were compared with those of the signature matrix of TICs to generate a proportion matrix for the TICs in GC tissues. We further applied Monte Carlo sampling algorithm to obtain a p value for the deconvolution of each sample, which providing a measure of confidence for the obtained data. A CIBERSORT p<0.05 were deemed qualified for further analysis. The levels of TICs in each GC sample were also calculated by using a single-sample gene set enrichment analysis (ssGSEA) algorithm.

### Identification and functional enrichment analysis of DEGs derived from different molecular subtypes

Package “limma” in R was used to identify DEGs between different cuproptosis molecular subtypes. A fold-change of 1.5 and an adjusted p-value of <0.05 were set up to screen DEGs. “ClusterProfiler”, “org.Hs.eg.db”, “enrichplot”, and “ggplot2” packages in R were used for gene ontology (GO) enrichment analysis of DEGs. Adjusted p value <0.05 was recognized to be statistically significant.

### Construction of CRG risk scoring system

CRG Risk scoring system was established to identify the cuproptosis patterns of the individual tumors. First, DEGs, screened out from distinct cuproptosis molecular subtypes, were subjected to univariate Cox regression analysis to seek those related to GC patients’ survival. Second, we divided patients into different cuproptosis gene subtype, including subtype A and B, through consensus clustering analysis based on the expression of prognostic DEGs. Third, “caret” package in R was used to randomly categorized all GC patients from TCGA-STAD and GSE84433 database into training (n=364) and testing (n=364) groups at a ratio of 1:1. Lastly, we constructed CRG Risk scoring system in the training group and further validated the system in the testing group and the combined group. In detail, we applied “glmnet” R package to conduct logistic least absolute shrinkage and selection operator (LASSO) Cox regression analysis to minimize the risk of over-fitting. The varied trajectory of each independent variable was analyzed and cross-validated to establish the model. Multivariate Cox analysis was applied again to seek candidate cuproptosis-related Risk genes and establish prognostic CRG Risk scoring system in the training set. The CRG Risk score was calculated as follows: CRG Risk score = Σ(Expi * coefi). Expi presented the expression of key cuproptosis-related Risk gene, and coefi presented the Risk coefficient. Correlation analysis was applied to evaluate the relationship between Risk score and different molecular or gene subtypes. A total of 364 GC patients in the training group were classified into high- (n=182) and low-risk (n=182) sets according to the median Risk score. Kaplan–Meier survival analysis was further used to identify the survival difference between high- and low-risk sets.

Similarly, both the testing and combined sets were grouped into high- and low-risk sets, each of which was subjected to survival analysis and the generation of receiver operating characteristic (ROC) curves.

### Tissue samples acquisition and real-time quantitative polymerase chain reaction (RT-qPCR)

Ten groups of GC and corresponding normal tissues were harvested from GC patients at Nanjing Jiangning Hospital. The study was permitted by the Ethics Committee of Nanjing Jiangning Hospital. RNA isolation and RT-qPCR were carried out as our previous description (31). The primer sequences used for qRT-PCR in this study are listed in **Table S3**.

### Evaluation of TME and different risk score groups

We identified the expression of CRGs in high- and low- Risk score groups through boxplots, and further calculated the abundance of TICs in TME of each GC sample by applying CIBERSORT in R. Correlation analyses were also carried out to study the relationships between TICs and prognostic Risk genes.

### Relationships of microsatellite instability (MSI), cancer stem cell (CSC), tumor mutational burden (TMB), and somatic mutations in distinct CRG risk score groups

We analyzed the associations of MSI, CSC, and TMB in two Risk groups. Mutation frequency analyses of high- and low- Risk groups were conducted by using the”maftools” R package.

### Drug susceptibility analysis

In order to investigate the therapeutic effects of drugs in the two groups, we calculated the semi-inhibitory concentration (IC50) values of drugs using “pRRophetic” package in R.

### Development of a nomogram scoring system

The clinicopathological features and CRG Risk score were incorporated to develop a nomogram using the “rms” package, based on patients’ survival. In the nomogram scoring system, a variable, such as gender, age, TNM stage and CRG Risk score, was matched with a score, and the total score was obtained by adding the scores across all variables of each sample. The subsequent calibration graph of the nomogram scoring system was performed to examine the predictive value between the predicted 1-, 3-, and 5-year survival rates and the virtually outcomes.

### Statistical analyses

All statistical analyses were performed using R version 4.2.1. Statistical significance was set at p < 0.05.

## Results

### Genetic and transcriptional alterations of CRGs in GC

**Figure 1:**
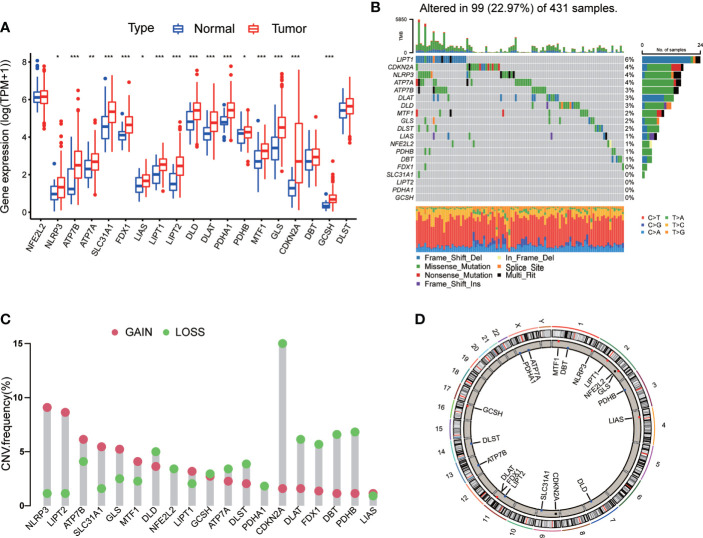
Genetic and transcriptional alterations of CRGs in GC. **(A)** The expression levels of 19 CRGs between 375 GC samples and 32 normal samples. Wilcoxon test was used to compare two groups. **(B)** The maftool exhibited incidence of somatic mutations of CRGs in 431 GC patients from TCGA database. **(C)** The CNV frequency of CRGs in 440 GC samples from TCGA database. **(D)** Locations of CNV alterations on 23 chromosomes. P < 0.05 was considered as significant importance. * indicated P < 0.05, ** indicated P < 0.01, *** indicated P < 0.001.

A total of 19 CRGs were analyzed in our following study, such as NFE2L2, NLRP3, ATP7B, ATP7A, SLC31A1, FDX1, LIAS and so on (**Table S2**). We first compared the expression of CRGs between tumor samples and normal samples from TCGA-STAD database, and found that 16 CRGs were up-regulated in tumor tissues, including NLRP3, ATP7A, ATP7B, SLC31A1, FDX1, LIAS, LIPT1, LIPT2, DLD, DLAT, PDHA1, PDHB, MTF1, GLS, CDKN2A, and GCSH (**Figure 1A**).

Next, we conducted general analysis of the somatic mutation frequency in these 19 CRGs, the result showed a relatively high mutation frequency in GC samples from TCGA-STAD database (**Figure 1B**). In detail, 99 (22.97%) GC samples had mutations in CRGs. LIPT1 had the highest mutation frequency (6%), followed by CDKN2A, NLRP3, ATP7A, ATP7B, DLAT, DLD, MTF1, GLS, DLST, LIAS, NFE2L2, PDHB and DBT. The other five CRGs, including FDX1, SLC31A1, LIPT2, PDHA1 and GCSH, did not have any mutations in tumor samples. Furthermore, we calculated somatic copy number alterations in these CRGs and found that copy number alterations were pervasive in all 19 CRGs. Among them, NLRP3, LIPT2, ATP7B, SLC31A1, GLS and MTF1 had relatively elevated copy number variation (CNV), while CDKN2A, DLAT, FDX1, DBT and PDHB showed relatively decreased CNV (**Figure 1C**). Detailed locations of CNV alterations on chromosomes were presented in **Figure 1D**.

From above, we noted that CRGs in GC tissues had prevalent genetic and transcriptional alterations, which might have their roles in GC oncogenesis.

### Identification of cuproptosis molecular subtypes

**Figure 2:**
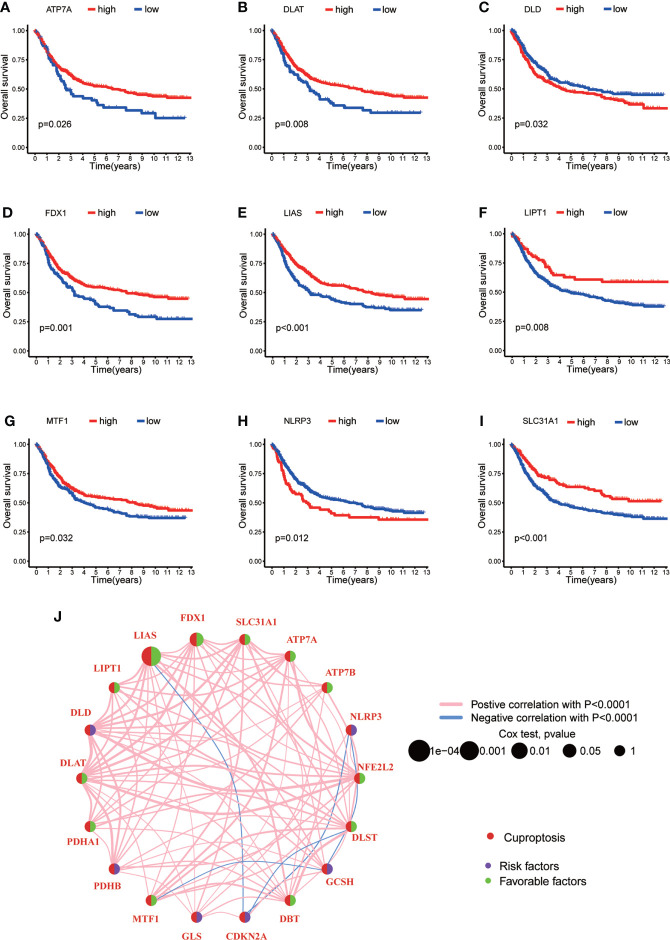
The survival analyses of CRGs and a comprehensive landscape of cuproptosis network in GC. **(A–I)** The survival analyses of CRGs (ATP7A, DLAT, DLD, FDX1, LIAS, LIPT1, MTF1, NLRP3 and SLC31A1) in 732 GC patients. Kaplan–Meier plot and log-rank tests were conducted for survival analyses. **(J)** Mutual correlations among CRGs in 732 GC samples. Spearman correlation analyses were used. The line between two CRGs indicated their interaction, and the stronger the correlation, the thicker the line. Pink line represented positive correlation and blue line represented negative correlation. P < 0.05 was considered to be statistically significant.

In order to explore the expression pattern of CRGs involved in GC tumorigenesis, we collected 732 GC patients from TCGA database (TCGA-STAD) and GEO database (GSE84433) for further analyses. Detailed clinicopathological information of patients was presented in **Table S1**. Survival analysis revealed that 9 CRGs (ATP7A, DLAT, DLD, FDX1, LIAS, LIPT1, MTF1, NLRP3 and SLC31A1) were significantly associated with overall survival (OS) of GC patients (**Figures 2A–I**, p<0.05). The result of univariate Cox regression analysis on CRGs showed that both LIAS and FDX1 were significantly associated with GC patients’ survival (**Table 1**). The intersection between survival analysis and multivariate Cox regression analysis indicated that both LIAS and FDX1 were significantly associated with the prognosis of GC patients. Next, a cuproptosis network was carried out to comprehensively demonstrate the association among CRGs and their prognostic value in GC patients (**Figure 2J**; **Table S4**). The network indicated there were prevalent and complicated interactions among CRGs.

**Table 1:** Multivariate Cox regression analyses of CRGs associated with OS in GC patients.

id	HR	HR.95L	HR.95H	P value
LIAS	0.628484	0.489251	0.8073397	0.000278
FDX1	0.778137	0.6304864	0.9603639	0.019455
SLC31A1	0.873637	0.7476713	1.0208252	0.089035
DLAT	0.862436	0.7240589	1.0272583	0.097202
MTF1	0.852843	0.6814416	1.0673578	0.16437
DBT	0.874791	0.7021722	1.0898458	0.232948
GCSH	1.196277	0.8872363	1.6129623	0.239867
LIPT1	0.860036	0.6513559	1.1355729	0.287622
PDHA1	0.898972	0.7362679	1.0976302	0.295787
DLD	1.077261	0.9031478	1.2849406	0.408008
NLRP3	1.069949	0.906117	1.2634027	0.425258
ATP7A	0.945946	0.7731582	1.1573494	0.589213
DLST	0.950347	0.7755852	1.1644879	0.623283
CDKN2A	1.015237	0.9438608	1.0920104	0.684323
PDHB	1.05097	0.8079387	1.3671055	0.711006
ATP7B	0.981223	0.8761234	1.0989298	0.742961
GLS	1.021636	0.8975885	1.1628266	0.745868
NFE2L2	0.991532	0.8153638	1.2057635	0.932099

**Figure 3:**
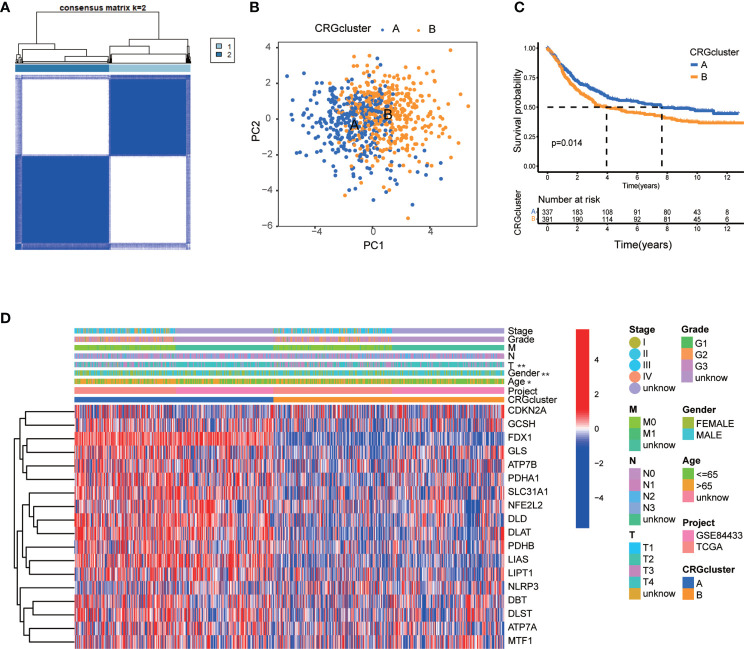
CRG molecular subtypes and their clinicopathological features. **(A)** Identification of two molecular subtypes (k = 2) and their correlation area through consensus clustering analysis in 732 GC samples. **(B)** PCA presented a great difference in transcriptomes between different molecular subtypes. **(C)** Survival analysis showed a significant difference of survival between molecular subtype A and (B) Kaplan–Meier plot and log-rank tests were conducted for survival analyses. **(D)** The heat-map showed the CRGs expression profile in molecular subtype A and B, and the associations between clinicopathologic characteristics and different molecular subtypes. Chi-square test was used for the comparison. Red color indicated up-regulated expression level and blue color indicated down-regulated expression level. P < 0.05 was considered to be statistically significant. Molecular subtype A contained 339 GC samples and molecular subtype B contained 393 GC samples. * indicated P < 0.05, ** indicated P < 0.01.

Regarding the comprehensive associations among CRGs, we categorized GC patients into two groups based on the expression profiles of CRGs by using a consensus clustering algorithm. The results indicated that k=2 might be an optimal selection for clarifying patients into 2 groups, including molecular subtype A (n=339) and B (n=393) (**Figure 3A**, **Figures S1A–I**; **Table S5**). PCA analysis verified that there were significant differences in the cuproptosis related transcription profiles between subtype A and B (**Figure 3B**). Survival analysis indicated that GC patients of subtype A had a higher survival probability than those in subtype B (log-rank test, p = 0.014; **Figure 3C**). In addition, the associations between CRGs expression and the clinical features, such as gender, age, grade and TNM stage, were profiled according to different molecular subtypes of GC (**Figure 3D**). The heat-map showed that a group of CRGs were upregulated in cluster A, such as FDX1, GLS, SLC31A1, LIAS and so on. And gender, age, and T stage were significantly correlated with GC patients’ cuproptosis subtypes (**Figure 3D**, p<0.05).

### Clinical features of TME in molecular subtype A and B

**Figure 4:**
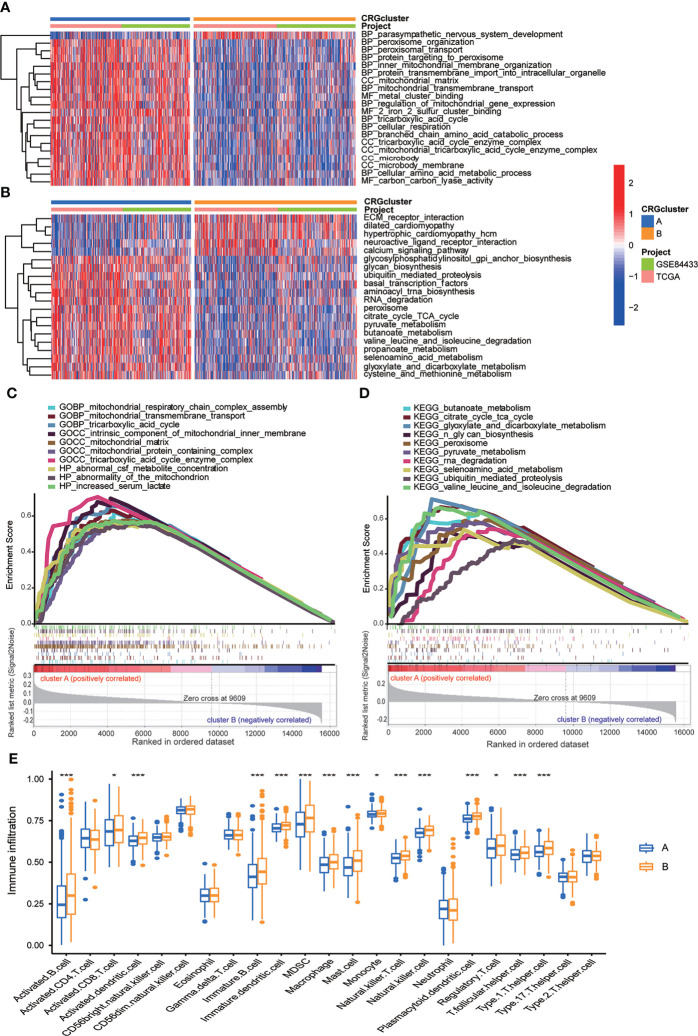
Correlations between TME and CRG molecular subtypes. **(A)** GO GSVA enrichment analyses between molecular subtype A and (B) Red color indicated more enriched in pathways and blue color indicated less enriched in pathways. Adjusted p value <0.05 was considered to be statistically significant. **(B)** KEGG GSVA enrichment analyses between molecular subtype A and (B) Red color indicated more enriched in pathways and blue color indicated less enriched in pathways. Adjusted p value <0.05 was considered to be statistically significant. **(C)** GO GSEA enrichment analyses of genes between molecular subtype A and (B) NES>1, nominal p value<0.05, FDR<0.25 were considered to be statistically significant. **(D)** KEGG GSEA enrichment analyses of genes between molecular subtype A and (B) NES>1, nominal p value<0.05, FDR<0.25 were considered to be statistically significant. **(E)** ssGSEA indicated differences between the infiltration levels of TICs and distinct molecular subtypes. P value<0.05 was considered to be statistically significant. Molecular subtype A contained 339 GC samples and molecular subtype B contained 393 GC samples. * indicated P < 0.05, *** indicated P < 0.001.

In order to further identify the characteristics of TME in distinct subtypes, we conducted not only GO and KEGG GSVA enrichment analysis, but also GSEA enrichment analysis. The results of GO GSVA enrichment analysis displayed that subtype A was significantly enriched in metabolic related pathways, including TCA cycle, peroxisomal organization and transportation, mitochondrial membrane organization and transportation, regulation of mitochondrial gene expression, protein transmembrane import, amino acid metabolic process, and so on (**Figure 4A**; **Table S6**). GSEA enrichment analysis suggested that for C5 collection, the gene ontology sets, genes in molecular subtype A were also enriched in the above metabolic related pathways (**Figure 4C**; **Table S7**). KEGG GSVA enrichment analysis also found that subtype A was primarily enriched in metabolic related pathways, such as TCA cycle, glycan biosynthesis, ubiquitin mediated proteolysis, peroxisome, RNA degradation, cysteine, methionine, glyoxylate, dicarboxylate, pyruvate, butanoate and selenoamino acid metabolism, valine leucine and isoleucine degradation and so on (**Figure 4B**; **Table S8**). GSEA enrichment analysis indicated that for C2 collection, the KEGG gene sets database, genes in molecular subtype A were also primarily enriched in the above metabolic related pathways (**Figure 4D**; **Table S9**). In particular, both GO and KEGG GSVA enrichment analysis found that TCA cycle, peroxisome, amino acid metabolism were obviously enriched in subtype A, and TCA cycle has been proven to be closely associated with cuproptosis (17).

We further explored whether CRGs were involved in TIME of GC through correlation analysis between two subtypes. Human immune cell subsets of each GC sample were calculated by using CIBERSORT algorithm (**Table S10**). We observed significant differences in the infiltration of most immune cells between subtype A and B through ssGSEA (**Figure 4E**). In detail, the infiltration levels of activated B cell, activated CD8 T cell, activated dendritic cell, immature B cell, immature dendritic cell, myeloid-derived suppressor cell (MDSC), macrophage, mast cell, monocyte, natural killer T cell, natural killer cell, plasmacytoid dendritic cell, regulatory T cell, T follicular helper cell and Type 1 T-helper cell were obviously lower in subtype A than those in subtype B (**Figure 4E**). From above, we primarily speculated that subtype A was enriched in metabolic related pathways, and subtype B was closely related with TIME.

### Identification of cuproptosis-related gene subtypes based on DEGs

**Figure 5:**
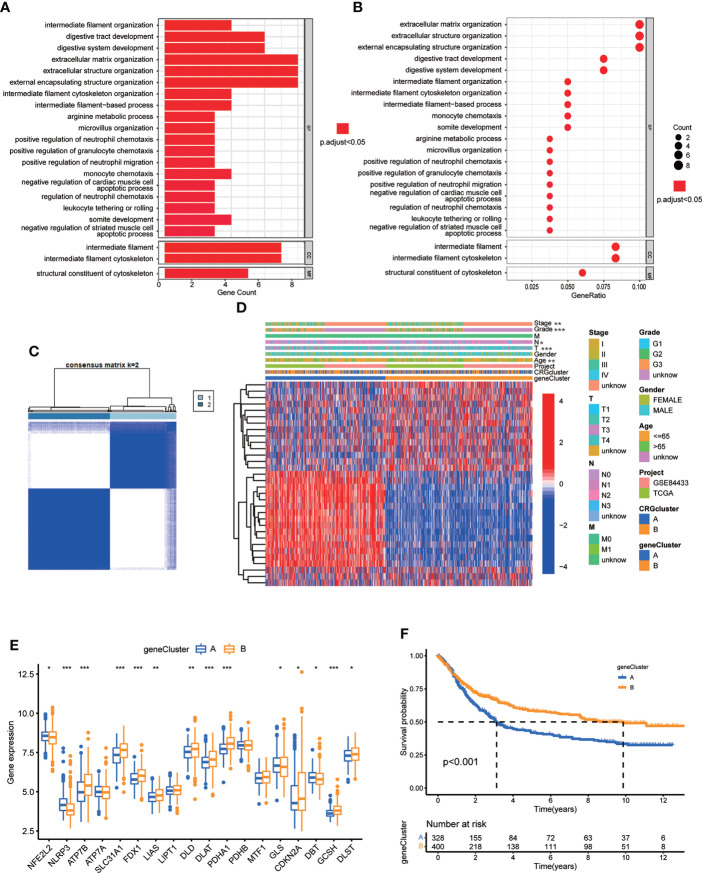
Identification of CRG gene subtypes based on 84 DEGs derived from different molecular subtypes. **(A, B)** GO enrichment analyses of 84 DEGs from molecular subtype A and (B) Adjusted p value<0.05 was considered to be statistically significant. **(C)** Identification of two gene subtypes (k = 2) and their correlation area through consensus clustering analysis according to the expression of 32 prognosis-related DEGs. **(D)** The heat-map showed the gene profiles in gene subtypes A and B, and the associations between clinicopathologic characteristics and distinct gene subtypes. Chi-square test was used for the comparison. P < 0.05 was considered to be statistically significant. **(E)** Differential analysis of the expression of CRGs in different gene subtypes. P < 0.05 was considered to be statistically significant. **(F)** Survival analysis of two gene subtypes. Kaplan–Meier plot and log-rank tests were conducted for survival analyses. P < 0.05 was considered to be statistically significant. Molecular subtype A contained 339 GC samples and molecular subtype B contained 393 GC samples. Gene subtype A and B contained 329 and 403 GC samples, respectively. * indicated P < 0.05, ** indicated P < 0.01, *** indicated P < 0.001.

To further investigate the underlying biological behavior of different cuproptosis molecular subtypes, we identified 84 DEGs between subtype A and B (**Table S11**). GO enrichment analysis was carried out to seek related biological pathways. The result showed that DEGs primarily enriched in digestive system development, extracellular matrix organization, extracellular structure organization, intermediate filament cytoskeleton and organization, arginine metabolic process and so on (**Figures 5A, B**; **Table S12**). According to the above enrichment analysis, we speculated that cuproptosis played an important role in the regulation of metabolism and intracellular/extracellular structure in GC. We further performed univariate Cox regression analysis to identify the prognostic value of 84 subtype-related DEGs and finally screened out 32 genes related to OS (p < 0.05), which were analyzed in the following section (**Table S13**)

Next, we performed consensus clustering analysis of 32 prognosis related DEGs to validate this regulation mechanism. Patients were divided into two genomic subtypes, namely, gene subtype A and B (**Figure 5C**; **Figure S2**; **Table S14**). The two cuproptosis gene subtypes presented significant differences in the expressions of CRGs, consistent with the expected results of the cuproptosis patterns (**Figure 5E**). In addition, cuproptosis gene subtype was significantly correlated with patients’ age, grade, and T and N stage (**Figure 5D**, p<0.05). The result of survival analysis showed that patients in gene subtype B had a higher survival probability than those in gene subtype A (**Figure 5F**, p<0.001).

### Construction and validation of prognostic CRG Risk scoring system

**Figure 6:**
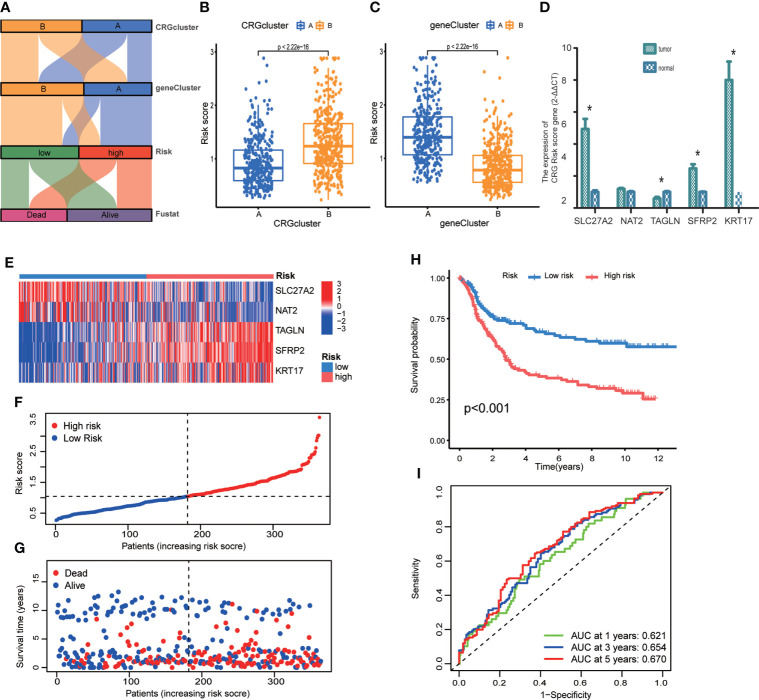
Construction of CRG Risk scoring system in the training group. **(A)** Alluvial diagram of subtype distributions in groups with different molecular subtypes, gene subtypes, Risk scores and survival outcomes. **(B)** Differential analysis of CRG Risk score in 339 molecular subtype A and 393 molecular subtype **(B, C)** Differential analysis of CRG Risk score in 329 gene subtype A and 403 gene subtype **(B, D)** RT-qPCR indicated the expression of five CRG risk score gene in 5 tumor and normal samples. * indicated P < 0.05. **(E)** Heat-map of five scoring genes expression profile in different risk sets of the training group. **(F, G)** Ranked dot and scatter plots of CRG Risk score distribution and patient survival in the training group. **(H)** Survival analysis in high- and low- CRG Risk score groups in the training set. Kaplan–Meier plot and log-rank tests were conducted for survival analyses. **(I)** ROC curve predicted the sensitivity and specificity of 1-, 3-, and 5-year survival according to CRG Risk score in the training group. The training group contained 364 GC samples. P < 0.05 was considered to be statistically significant.

We established CRG Risk scoring system according to the expression of prognostic DEGs derived from distinct molecular subtypes. As shown in **Figure 6A**, GC patients were grouped into two cuproptosis molecular subtypes, two gene subtypes, and two CRG Risk score groups. First, we randomly classified GC patients into training (n=364) and testing (n=364) groups at a ratio of 1:1, by using “caret” package in R (**Tables S15**, **16**). LASSO and multivariate Cox analyses of 32 prognostic DEGs were used to seek optimum prognostic signature (**Figure S3**). Then multivariate Cox regression analysis was carried out to construct CRG Risk scoring system in the training sets: Risk score = (-0.145406843672574 * expression of SLC27A2) + (-0.100834783610163 * expression of NAT2) + (0.108030183332151* expression of TAGLN) + (0.0800602829915149* expression of SFRP2) + (0.0876442662604965 * expression of KRT17). We calculated Risk score of each GC samples in both molecular subtypes and gene subtypes, and found that Risk score was significantly elevated in molecular subtype B and gene subtype A, compared with that in molecular subtype A and gene subtype B, respectively (**Figures 6B, C**). Next, the expressions of five key cuproptosis-related risk genes in the training sets were profiled in **Figure 6E**, based on CRG Risk score (**Table S17**). The results indicated that the expression of five key Risk scoring genes showed a great difference between high- and low- risk sets (**Figure 6E**). The levels of five cuproptosis-related Risk genes were also measured in GC tissues and adjacent normal tissues by RT-qPCR. As shown in **Figure 6D**, the expression of SLC27A2, SFRP2, KRT17 were up-regulated in tumor tissues (p<0.05), the expression of TAGLN was down-regulated (p<0.05), while those of NAT2 remained unchanged (p>0.05), compared with the levels in the corresponding normal tissues.

The scattergram of CRG Risk score in the training sets revealed that patients’ survival time decreased while CRG Risk score increased (**Figures 6F, G**). The Kaplan–Meier survival curves showed that GC patients with low Risk scores had a better overall survival compared to that in patients with high Risk scores (**Figure 6H**, p <0.001). Additionally, the 1-, 3-, and 5-year survival rates of CRG Risk score were represented by area under the time-concentration curve (AUC) values of 0.621, 0.654, and 0.670, respectively, indicating both moderate sensitivity and specificity (**Figure 6I**). The CRG Risk score predicted 1- year survival with a 70% specificity and 50% sensitivity, 3- year survival with a 42% specificity and 83% sensitivity, and 5-year survival with a 61% specificity and 65% sensitivity.

To validate the accuracy of the CRG Risk scoring system, we calculated CRG Risk score in the testing group, and combined TCGA-STAD and GSE84433 group (**Figures S4**, **S5**; **Tables S18**, **19**). GC patients were stratified into high- and low-risk sets, the same as which in the training set. The expression of five key Risk scoring genes in the testing set and the combined set, were presented in **Figure S4A** and **S5A**, respectively. The relationships between patients’ survival and CRG Risk score were shown in **Figures S4B, C**, **S5B, C**. Survival analyses presented that GC patients with low CRG Risk scores had a significantly favorable overall survival compared to those in patients with high scores, which was the same in the training group (**Figure S4D**, p=0.002; **Figure S5D**, p<0.001). We also conducted prognostic prediction classification efficiency analysis and found that CRG Risk score still had relatively high AUC values (**Figures S4E**, **S5E**), suggesting that the CRG Risk scoring system was suitable to accurately predict the survival of GC patients.

### Relationships between TME and different groups of CRG risk score

**Figure 7:**
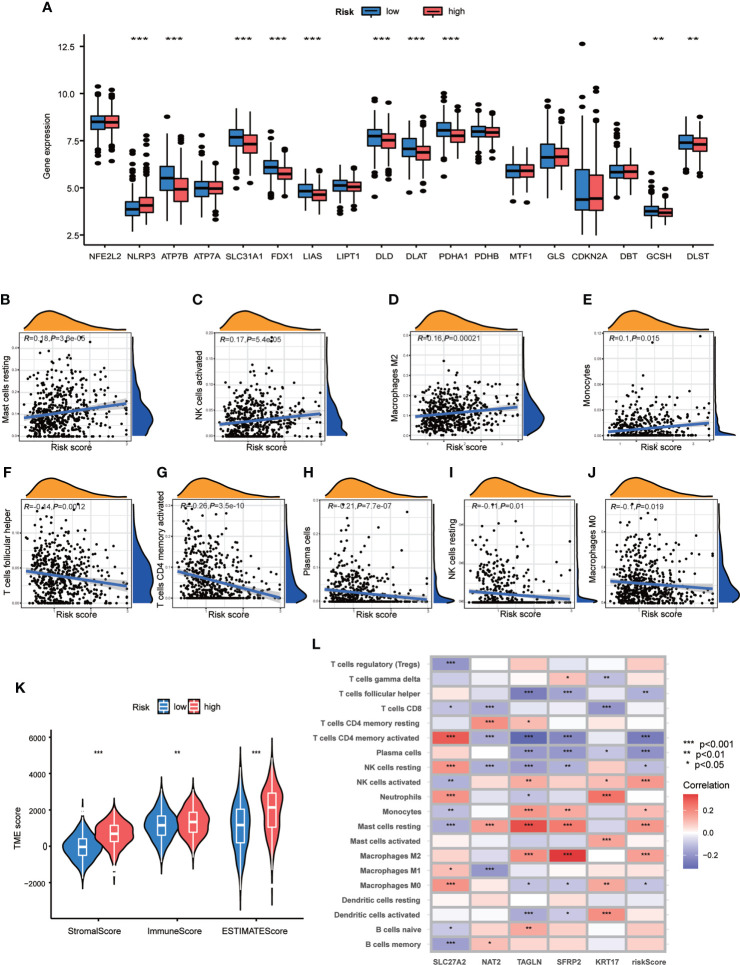
Associations of TME and CRG Risk score. **(A)** Differential analyses of CRGs expression in the high- and low-risk score groups. **(B–J)** Correlation analyses between CRG Risk score and TICs. **(K)** Differential analyses between CRG Risk score and immune/stromal/estimate scores. **(L)** Correlation analyses between the abundance of TICs and five key Risk scoring genes in the proposed model. High-risk score group contained 352 GC samples and low-risk score group contained 376 GC samples. P < 0.05 was considered to be statistically significant. * indicated P < 0.05, ** indicated P < 0.01, *** indicated P < 0.001.

In order to explore the relationship of TME and CRG Risk score, we first analyzed the expression of CRGs in both high- and low- CRG Risk score groups, and found that ten CRGs were significantly related with CRG Risk score. To be specific, ATP7B, SLC31A1, FDX1, LIAS, DLD, DLAT, PDHA1, GCSH and DLST were down-regulated in high-Risk score group, while NLRP3 were up-regulated in high-Risk score group (**Figure 7A**). Next, we performed correlation analyses to clarify the relationship between the abundance of immune cells and CRG Risk score, through applying CIBERSORT algorithm. As shown in **Figures 7B–J**, CRG Risk score was positively related with resting Mast cells, activated natural killer (NK) cells, M2 Macrophages and monocytes, while negatively associated with follicular helper T cells, activated memory CD4 + T cells, plasma cells, resting NK cells and M0 macrophages. A high CRG Risk score was positively associated with both immune and stromal score (**Figure 7K**). In addition, we studied the association between the abundance of immune cells and the five key cuproptosis-related Risk genes. The results showed that the majority of immune cells were significantly related with the five genes (**Figure 7L**). As a result, CRG Risk score may be related with tumor TIME of GC.

### Association of CRG risk score with MSI, CSC, TMB, and somatic mutations

**Figure 8:**
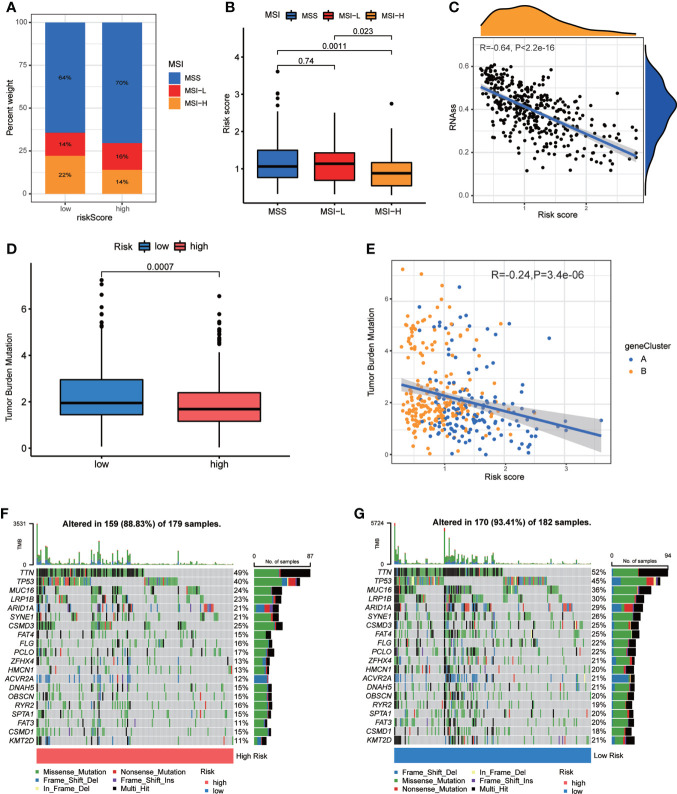
Associations of CRG Risk score with MSI, TMB and CSC. **(A)** The distribution of MSI in different Risk score groups. **(B)** Differential analyses between CRG Risk score and MSI. **(C)** Correlation analysis between CRG Risk score and CSC index. **(D)** Differential analysis of TMB in distinct CRG Risk score groups. **(E)** Correlation analysis of CRG Risk score and TMB. **(F, G)** The waterfall plot of somatic mutation characteristics in high- and low- CRG Risk score groups. High-risk score group contained 352 GC samples and low-risk score group contained 376 GC samples. P < 0.05 was considered to be statistically significant.

Accumulating evidence has implied that MSI is a potential genomic biomarker to identify patients’ sensitivity to immunotherapy (32). We assessed the MSI status in distinct sets of CRG Risk score. In the high-score group, MSI-H accounted for 14%, MSI-L accounted for 16%, the rest 70% were microsatellite stable (MSS) (**Figure 8A**). However, in the low-score group, the proportion of MSI-H was significantly increased (22%), and the proportion of MSI-L was decreased (14%) (**Figure 8A**). Further correlation analyses indicated that a low CRG score was significantly related with MSI-H status, while a high CRG score was correlated with MSS status (**Figure 8B**). This might be associated with better efficacy of immunotherapy.

CSCs have been recognized as promising therapeutic targets for cancer therapy according to their self-renewal capacity and differentiation potential (33). As a result, we studied the correlation between CRG Risk score and CSC index values. **Figure 8C** showed the results of the negative linear correlation between CRG Risk score and CSC index (R = −0.64, p <.001), suggesting that GC cells with low CRG Risk score had more different stem cell properties and a lower degree of cell differentiation (**Figure 8C**).

TMB, reflecting cancer mutation quantity, is also clinically related with immune checkpoint inhibitors (ICIs) outcomes (34). Patients with a higher TMB usually benefited from ICIs. Our analysis of the mutation data from TCGA-STAD cohort demonstrated that lower TMB was observed in the sets of high CRG Risk score than that in the sets of low CRG Risk score (p<0.001; **Figure 8D**). Spearman correlation analysis discovered that TMB was negatively associated with CRG Risk score (R = −0.24, p = 3.4e-06; **Figure 8E**). The above analyses indicated that the low-risk set might benefit from ICIs. We further described the distribution variations of the somatic mutations between two CRG Risk score sets in TCGA-STAD cohort through maftools. The top ten mutated genes in the high- and low-CRG Risk sets were TTN, TP53, MUC16, LRP1B, ARID1A, SYNE1, CSMD3, FAT4, FLG, PCLO and ZFHX4 (**Figures 8F, G**). Patients with a low CRG Risk score had obviously higher frequencies of all these mutations, except CSMD3, compared to those in patients with a high CRG Risk score.

### Drugs susceptibility analysis

To investigate the therapeutic effects of drugs in patients of the two groups, we applied “pRRophetic” package in R to calculate the IC50 values of drugs. We assessed not only the current clinical used chemotherapy drugs, but also drugs under clinical trials. We classified drugs into multiple groups, including AKT inhibitors, BCL-2 inhibitors, PI3K inhibitors, MEK inhibitors, ROCK inhibitors, XIAP inhibitors, Raf inhibitors, Multikinase inhibitors, and so on. Interestingly, we found that the patients in the low CRG Risk score group had higher IC50 value for AKT inhibitors (MK.2206), ALK inhibitor (NVP.TAE684), proteasome inhibitor (Bortezomib, MG.132), ATP inhibitor (PF.562271), Bcl-2 inhibitor (ABT.263, TW.37), Bcr-Abl inhibitor (Imatinib, GNF.2), BTK inhibitor (LFM.A13), CDK inhibitor (CGP.60474, PD.0332991), Chk1 inhibitor (AZD7762), DNA synthesis inhibitor (Bleomycin), FGFR inhibitor (PD.173074), FTase inhibitor (FTI.277), GSK-3 inhibitor (CHIR.99021, SB.216763), HIF-PH inhibitor (DMOG), IGF-1RIR inhibitor (BMS.536924, BMS.754807), ITK inhibitor (BMS.509744), JNK inhibitor (AS601245, JNK.9L, JNK.Inhibitor.VIII), PKC Modulator (Bryostatin.1, Midostaurin), MEK inhibitor (RDEA119), MET inhibitor (PF.02341066, PHA.665752), mTOR inhibitor (Temsirolimus, AZD8055), mTOR and PI3K inhibitor (NVP.BEZ235), ATM inhibitor (KU.55933), Bcr-Abl inhibitor (Nilotinib), Doxorubicin, Elesclomol, Docetaxel, Lck/Src inhibitor (KIN001.135), MDM2 inhibitor (JNJ.26854165), PDK1 inhibitor (BX.795), PI3K inhibitor (AZD6482, GDC0941), PK inhibitor (NU.7441), RXR activator (Bexarotene), VEGFR inhibitor (AMG.706), ROCK inhibitor (GSK269962A), RSK inhibitor (CMK), Src inhibitor (A.770041, AZD.0530, WH.4.023), WIP1 inhibitor (CCT007093) and XIAP inhibitor (Embelin). However, patients in the low CRG Risk score group had lower IC50 value for eIF2α dephosphorylation inhibitor (Salubrinal). methotrexate, TrkA inhibitor (GW.441756), TNF inhibitor (Lenalidomide), PLK inhibitor (GW843682X), Rac1 inhibitor (EHT.1864), Aurora inhibitor (VX.680), Mitomycin.C, DHFR inhibitor (Pyrimethamine), RSK inhibitor (PF.4708671) and HDAC inhibitor (Vorinostat, MS.275). Furthermore, the same type of drugs might act different roles in different Risk score groups. For instance, patients in the low CRG Risk score group had higher IC50 value for Raf inhibitor (PLX4720, AZ628), and lower IC50 value for Raf inhibitor (SB590885). In the low CRG Risk score group, MAPK inhibitor (VX.702) displayed a higher IC50 value, while MAPK inhibitor (BIRB.0796) showed a lower IC50 value. Multikinase inhibitors (Dasatinib, Pazopanib, AP.24534, CEP.701) exhibited a better drug susceptibility in the group of low CRG Risk score, while multikinase inhibitor (Sorafenib) showed the opposite effect. The opposite drug susceptibility in low and high CRG Risk score groups were also seen between PARP inhibitor AZD.2281, AG.014699 and ABT.888, EGFR/Her-1/2 inhibitor Lapatinib and BIBW2992, HSP90 inhibitor AUY922 and CCT018159. Together, these results showed that CRGs were significantly related to drug sensitivity (**Figures S6**, **7**; **Table 2**).

**Table 2:** The drug susceptibility in patients of low- and high- score groups.

Drugs		Low-score group	High-score group
AKT inhibitors	MK.2206	+	–
ALK inhibitor	NVP.TAE684	+	–
proteasome inhibitor	Bortezomib	+	–
	MG.132	+	–
ATP inhibitor	PF.562271	+	–
Bcl-2 inhibitor	ABT.263	+	–
	TW.37	+	–
Bcr-Abl inhibitor	Imatinib	+	–
	GNF.2	+	–
BTK inhibitor	LFM.A13	+	–
CDK inhibitor	CGP.60474	+	–
	PD.0332991	+	–
Chk1 inhibitor	AZD7762	+	–
DNA synthesis inhibitor	Bleomycin	+	–
FGFR inhibitor	PD.173074	+	–
FTase inhibitor	FTI.277	+	–
GSK-3 inhibitor	CHIR.99021	+	–
	SB.216763	+	–
HIF-PH inhibitor	DMOG	+	–
IGF-1RIR inhibitor	BMS.536924	+	–
	BMS.754807	+	–
ITK inhibitor	BMS.509744	+	–
JNK inhibitor	AS601245	+	–
	JNK.9L	+	–
	JNK.Inhibitor.VIII	+	–
PKC Modulator	Bryostatin.1	+	–
	Midostaurin	+	–
MEK inhibitor	RDEA119	+	–
MET inhibitor	PF.02341066	+	–
	PHA.665752	+	–
mTOR inhibitor	Temsirolimus	+	–
	AZD8055	+	–
mTOR and PI3K inhibitor	NVP.BEZ235	+	–
ATM inhibitor	KU.55933	+	–
Bcr-Abl inhibitor	Nilotinib	+	–
	Doxorubicin	+	–
	Elesclomol	+	–
	Docetaxel	+	–
Lck/Src inhibitor	KIN001.135	+	–
MDM2 inhibitor	JNJ.26854165	+	–
PDK1 inhibitor	BX.795	+	–
PI3K inhibitor	AZD6482	+	–
	GDC0941	+	–
PK inhibitor	NU.7441	+	–
RXR activator	Bexarotene	+	–
VEGFR inhibitor	AMG.706	+	–
ROCK inhibitor	GSK269962A	+	–
RSK inhibitor	CMK	+	–
Src inhibitor	A.770041	+	–
	AZD.0530	+	–
	WH.4.023	+	–
WIP1 inhibitor	CCT007093	+	–
XIAP inhibitor	Embelin	+	–
eIF2α Dephosphorylation inhibitor	Salubrinal	–	+
TrkA inhibitor	GW.441756	–	+
TNF inhibitor	Lenalidomide	–	+
PLK inhibitor	GW843682X	–	+
Rac1 inhibitor	EHT.1864	–	+
Aurora inhibitor	VX.680	–	+
	Mitomycin.C	–	+
DHFR inhibitor	Pyrimethamine	–	+
RSK inhibitor	PF.4708671	–	+
HDAC inhibitor	Vorinostat	–	+
	MS.275	–	+
Raf inhibitor	PLX4720	+	–
	AZ628	+	–
	SB590885	–	+
MAPK inhibitor	VX.702	+	–
	BIRB.0796	–	+
multikinase inhibitor	Dasatinib	+	–
	Pazopanib	+	–
	AP.24534	+	–
	CEP.701	+	–
	Sorafenib	–	+
PARP inhibitor	AZD.2281	+	–
	AG.014699	+	–
	ABT.888	–	+
EGFR/Her-1/2 inhibitor	Lapatinib	+	–
	BIBW2992	–	+
HSP90 inhibitor	AUY922	+	–
	CCT018159	–	+

"+":indicated up-regulated sensitivity; "-":indicated down-regulated sensitivity.

### Establishment of a nomogram to predict GC patients’ survival

**Figure 9:**
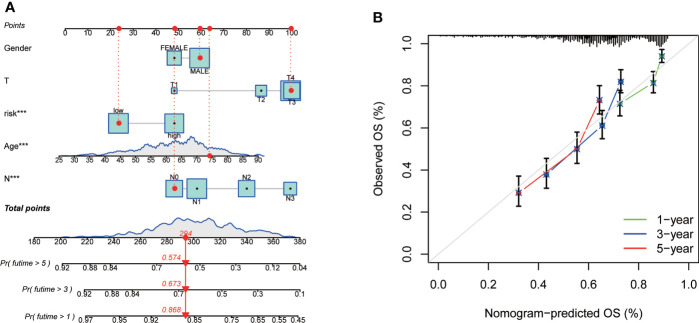
Construction and validation of a nomogram in 728 GC samples. **(A)** Nomogram for predicting the 1-, 3-, and 5-year OS of GC patients. **(B)** Calibration curves of the nomogram. 728 GC samples contained 352 high-risk score and 376 low-risk score.

As the importance of CRG Risk score in GC patients’ survival, we established a nomogram incorporating the CRG Risk score and clinicopathological features to predict the 1-, 3-, and 5-year OS rates (**Figure 9A**). Clinicopathological features contained gender, age and TNM stage. The subsequent calibration graph indicated that the proposed nomogram had a similar performance in GC patients compared to an ideal model (**Figure 9B**).

## Discussion

Gastric cancer is a global health problem. Despite the incidence and mortality decline over the past 5 decades, gastric cancer remains the third leading cause of cancer death worldwide (35). The clinical efficacy of conventional chemotherapy is limited, and the survival of advanced GC remains poor (36). Pioneer researches indicate that risk factors for GC are involved in the interplay between genetic susceptibility and environmental exposure (2). Based on the exploration of TME, ICIs, a kind of monoclonal antibodies that inhibit programmed cell death protein 1 (PD-1), PD-L1, and cytotoxic T-lymphocyte antigen 4 (CTLA-4), emerged as an exciting treatment strategy across a variety of malignancies in the last decade (37). TMB, MSI, PD-L1 and Epstein-Barr virus are recognized as potential biomarkers to identify susceptibility to ICIs. However, the number of patients benefit from ICIs is limited, and the primary and acquired resistance remains a big problem. Therefore, a comprehensive understanding of the alterations in the genome, transcriptome, and somatic mutation in TME is ultimate for the prevention, treatment, and prognosis evaluation of GC.

RCD, also known as programmed cell death (PCD), is generally regulated by genetic reprogramming of the cell that leads to an energy-dependent cascade of biochemical and morphological changes (38). Numerous researches have revealed that RCD takes a great part in kinds of pathological and physiologic processes, including tumorigenesis (39). An increasing number of novel forms of RCD (apoptosis, necroptosis, autophagy, ferroptosis, pyroptosis, alkaliptosis, oxeiptosis, parthanatos, entotic cell death, netotic cell death, and lysosome-dependent cell death) have been identified and are being comprehensively studied in various cancers (40). For example, ferroptosis, a novel iron-dependent lipid peroxidation induced RCD, was recently identified and found to be greatly involved in tumorigenesis (41). Evaluation of the prognostic value of ferroptosis-related genes has been widely performed in various tumors, such as pancreatic cancer, bladder cancer, colon cancer, hepatocellular carcinoma, glioblastoma, soft tissue sarcoma, thyroid papillary carcinoma, gastric cancer, ovarian cancer and so on (42–50). Associations between ferroptosis-related genes and TME have also been investigated in multiple cancers, including breast cancer, hepatocellular carcinoma, clear cell renal cell carcinoma, lung adenocarcinoma, head and neck squamous cell carcinoma, melanoma, papillary thyroid carcinoma and so on (51–57). Classifying tumor patients into distinct subtypes based on their molecular characteristics, enables us to better predict distinct phenotypes, drugs susceptibility, and prognosis of cancer patients (58).

Copper is the 26th element in abundance on earth (59). Aberrant copper homeostasis (ACH) are probably associated to metabolic activity, because copper is a cofactor for cytochrome C oxidase, which is involved in the electron transport chain (60). As an important trace element, despite the role of copper in GC has been primarily studied, it remains contradictory. For example, serum copper level was elevated in GC patients and significantly correlated to the survival time (61). Serum Cu: Zn ratio was also elevated, especially in advanced GC (62). Some studies focused on copper/zinc-superoxide dismutase (Cu/Zn-SOD) and found that serum levels of Cu/Zn SOD were significantly up-regulated in GC and higher Cu/Zn SOD levels indicated an increased risk of GC (63). Another research payed attention to Cu/Zn-SOD immunoreactivity and found that Cu/Zn-SOD was widely distributed in the gastric mucosa and the grade of Cu/Zn-SOD immunoreactivity was greatly associated with the histological type of GC, suggesting the function of GC cells may be vulnerable to active oxygen species. Additionally, well-differentiated gastric cancer appeared to be more frequently positive (64). However, Bo, L.Y.et.al (65). discovered that elevated level of Cu^2+^ was associated with higher growth inhibition, cell cycle arrest, mitochondrial membrane potential disruption, autophagy inhibition, and apoptosis induction. In addition, disulfiram (DSF) was an approved drug for anti-alcoholism medication and accumulating evidence suggested DSF, in combination with copper, showed excellent antitumor activity in multiple cancers, including GC (66). For example, DSF/Cu complex exhibited antitumor activity against GC cells *via* modulating the stress response, glycolysis, S6K1, c-Myc and Wnt/β-catenin signaling (67, 68). DSF/Cu also induced apoptosis through reactive oxygen species (ROS)/mitogen-activated protein kinase pathway (69). However, the role of cuproptosis in GC remains unknown, and the evaluation of prognostic value of CRGs has never been conducted in GC.

Luckily, the large-scale public database, including TCGA and GEO database, allows us to access and analysis the transcriptome profiles of multiple cancers, thus we can have an overall view of the genetic landscape, screen potential biomarkers, develop treatment strategies and predict patients’ prognosis (70, 71). In our study, we downloaded transcriptome profiling and the corresponding clinicopathological data of GC samples from TCGA and GEO databases. Firstly, we examined the levels of CGRs in tumor and normal tissues, and found that most CRGs were obviously elevated in tumor tissues. General analyses of somatic mutation frequency and copy number alterations in these 19 CRGs exhibited a relatively high mutation frequency and copy number alterations in GC samples. Survival analysis and univariate Cox regression analysis of GC patients from TCGA-STAD and GSE84433 databases revealed that a set of CRGs were significantly correlated with GC patients’ survival. Therefore, we hypothesized cuproptosis might be a potential target for the treatment of GC, and CRGs signature might serve to predict therapeutic response and prognosis of GC patients, which provided us new sights for exploring the role of copper in GC. As a result, we further grouped GC patients into molecular subtype A and B based on the expression profile of CRGs. Survival analysis showed that GC patients in subtype A had a higher survival probability than those in subtype B. Gender, age and T stage were also significantly associated with different subtypes. GSVA enrichment analysis indicated that subtype A was obviously enriched in metabolic related pathways, which was consistent with the findings of Tsvetkov et al. (17). Considering the important position of immunotherapy in gastric cancer, we further evaluated TIME related indicators, such as TICs, MSI, CSC, TMB and somatic mutations, to better explain the association between CRGs and TIME of GC. The profiling of TICs showed that most immune cells were more enriched in subtype B than in subtype A. GO and KEGG enrichment analyses of DEGs obtained from subtype A and B showed that these DEGs took a great part in the regulation of metabolism. Univariate Cox regression analysis was utilized to seek prognosis related DEGs. Then, patients were again divided into two sets (gene subtype A and B), based on the expression of 32 prognostic DEGs. Different gene subtypes showed great differences in CRGs, and were significantly correlated with patients’ age, grade, T and N stage, and survival. Regarding the great importance of CRGs in the prognosis of GC, we further established CRG Risk scoring system based on the expression of prognostic DEGs in the training group. The accuracy of system was validated in the testing group and the combined group. Patients with high Risk score showed a poorer survival rate than those with low Risk score. Furthermore, all of CGRs, TICs, MSI, CSC, TMB, somatic mutations, and drugs susceptibility were significantly related with different Risk score groups. Finally, we established a nomogram incorporating the CRG Risk score and clinicopathological features to predict 1-, 3-, and 5-year OS rates of GC patients. CRG Risk score was previously reported by Jiang R et al. (21) in ESCA and Wang et al. (72) in HCC. A high cuproptosis-related risk score was correlated with poor survival and pro-tumor immune infiltrates in TME of HCC and ESCA. The Risk score in ESCA was based on the expression of six CRGs (SLC25A5, SLC23A2, PDHX, COX7B, ATP7A and PIH1D2). However, it was different in HCC, and the Risk score was calculated by the expression of distinct five CRGs. We speculated that the unique TME determined the uniqueness and importance of CRGs Risk scoring system in each tumor. It is not appropriate to apply CRG Risk score derived from ESCA or HCC to evaluate the characteristics of TME, or predict survival and therapeutic response in GC. As a result, we established our own CRG Risk score system based on the comprehensive analyses of TME and evaluated its application in GC. However, our research on the relationship between CRG and TME of GC were almost based on the bioinformatics analysis. Further *in vitro* and *in vivo* experiments are required to explore the specific mechanism of CRGs affecting TME, which might be extremely important in the treatment of GC.

In conclusion, CRGs were significantly associated with TME, drugs susceptibility, prognosis evaluation of GC. The CRGs Risk scoring system served to accurately predict GC patient survival. Patients with a high CRGs Risk score showed shorter survival time, and less MSI, CSC and TMB. In addition, CRGs Risk scoring system exhibited a good capability in predicting the patients’ responsiveness to specific therapeutic drugs.

## Data availability statement

The original contributions presented in the study are included in the article/**Supplementary Material**. Further inquiries can be directed to the corresponding author.

## Ethics statement

The studies involving human participants were reviewed and approved by Nanjing Jiangning Hospital. The patients/participants provided their written informed consent to participate in this study.

## Author contributions

(I) Conception and design: JW, BW, and JC. (II) Administrative support: XH, ZT, and BL. (III) Provision of study materials or patients: JW, DQ, and XH. (IV) Collection and assembly of data: JW and YX. (V) Data analysis and interpretation: JW and SZ. (VI) Manuscript writing: JW and YW. (VII) Final approval of manuscript: All authors

## Funding

This research was funded by the National Nature Science Foundation of China, grant number 82103032, Medical Research Grant of Jiangsu Commission of Health, grant number M2020010, the Medical Science and Technology Development Foundation of Nanjing, grant number YKK21224.

## Conflict of interest

The authors declare that the research was conducted in the absence of any commercial or financial relationships that could be construed as a potential conflict of interest.

## Publisher’s note

All claims expressed in this article are solely those of the authors and do not necessarily represent those of their affiliated organizations, or those of the publisher, the editors and the reviewers. Any product that may be evaluated in this article, or claim that may be made by its manufacturer, is not guaranteed or endorsed by the publisher.

## Supplementary material

Supplementary Figure 1 Unsupervised clustering of CRGs and consensus matrix heat-maps for k = 3-9 through consensus clustering analysis in 732 GC samples.

Supplementary Figure 2 Unsupervised clustering of prognostic genes and consensus matrix heat-maps for k = 3-9 through consensus clustering analysis in 732 GC samples.

Supplementary Figure 3 Identification of optimum prognostic genes in 732 GC samples. **(A, B)** The LASSO regression analysis and partial likelihood deviance analysis on 32 subtype-related prognostic DEGs.

Supplementary Figure 4 Validation of CRG Risk score in the testing group. **(A)** The heat-map of five scoring genes expression in different risk sets of the testing group. **(B, C)** Ranked dot and scatter plots of CRG Risk score distribution and patient survival in the testing group. **(D)** Survival analysis of high- and low- CRG Risk score in the testing group. Kaplan–Meier plot and log-rank tests were conducted for survival analyses. P < 0.05 was considered to be statistically significant. **(E)** ROC curve predicted the sensitivity and specificity of 1-, 3-, and 5-year survival according to CRG Risk score in the testing group. The testing group included 364 GC samples, among which 170 samples were in high-risk group and 194 samples were in low-risk group.

Supplementary Figure 5 Validation of CRG Risk score in the combined TCGA-STAD and GSE84433. **(A)** The heat-map of the expression profile of five scoring genes in different risk groups. **(B, C)** Ranked dot and scatter plots of CRG Risk score distribution and patient survival in the combined group. **(D)** Survival analysis of high- and low- CRG Risk score in the combined group. Kaplan–Meier plot and log-rank tests were conducted for survival analyses. P < 0.05 was considered to be statistically significant. **(E)** ROC curve predicted the sensitivity and specificity of 1-, 3-, and 5-year survival according to CRG Risk score in the combined group. Combined TCGA-STAD and GSE84433 included 728 GC sample, among which 352 GC samples were in high-risk score group and 376 GC samples were in low-risk score group.

Supplementary Figure 6 Differential drugs susceptibility analyses in high- and low- Risk group. High-risk score group contained 352 GC samples and low-risk score group contained 376 GC samples. P < 0.05 was considered to be statistically significant.

Supplementary Figure 7 Differential drugs susceptibility analyses in high- and low- Risk group. High-risk score group contained 352 GC samples and low-risk score group contained 376 GC samples. P < 0.05 was considered to be statistically significant.
