# Supplementary figures and images for: Identification of cuproptosis-related subtypes, construction of a prognosis model, and tumor microenvironment landscape in gastric cancer
Source: Front Immunol. 2022 Nov 21;13:1056932. doi: 10.3389/fimmu.2022.1056932 (PMC9719959; doi:10.3389/fimmu.2022.1056932)

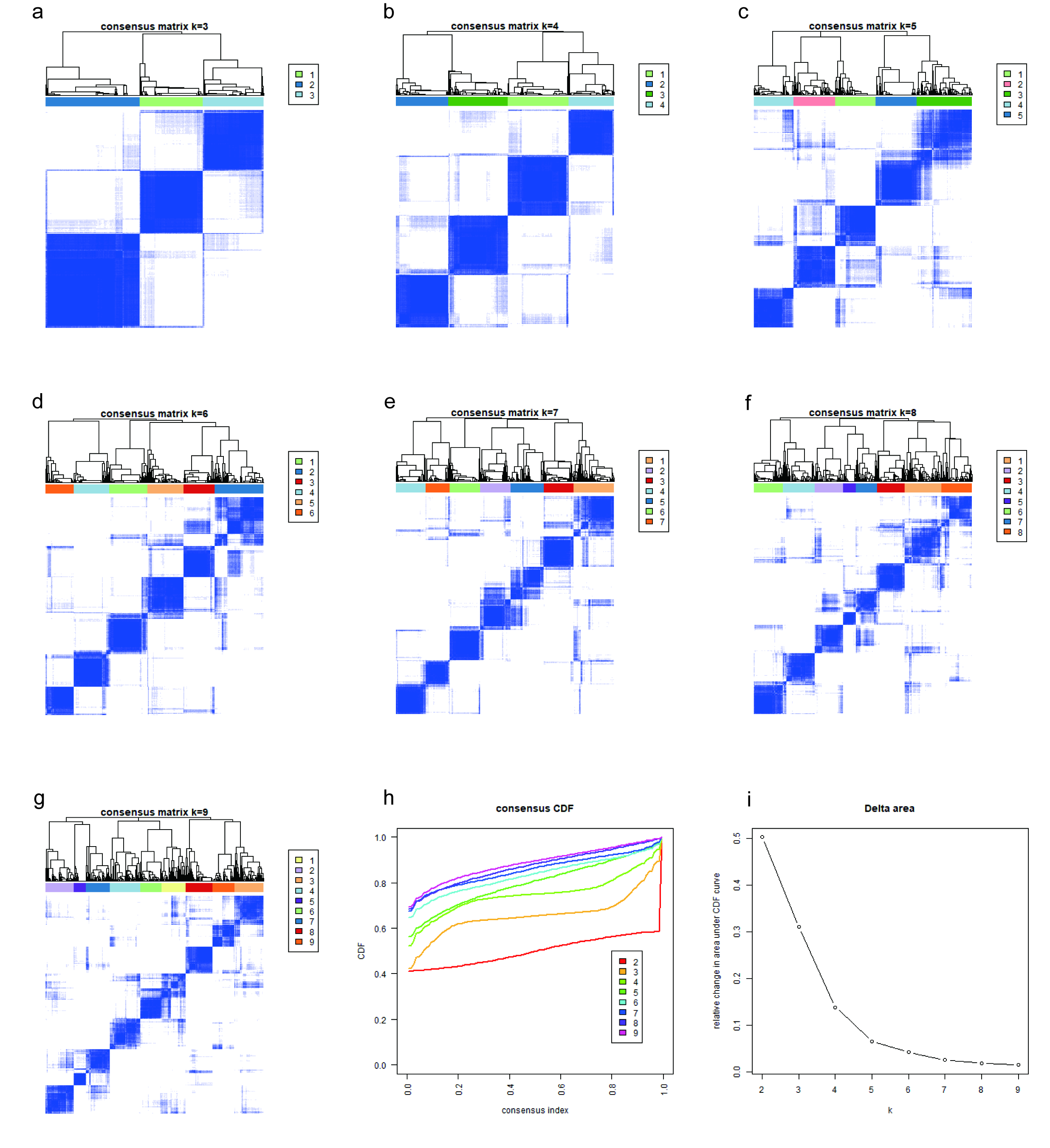

Supplement: Supplementary Figure 1 — Unsupervised clustering of CRGs and consensus matrix heat-maps for k = 3-9 through consensus clustering analysis in 732 GC samples. [file Image_1.tif]

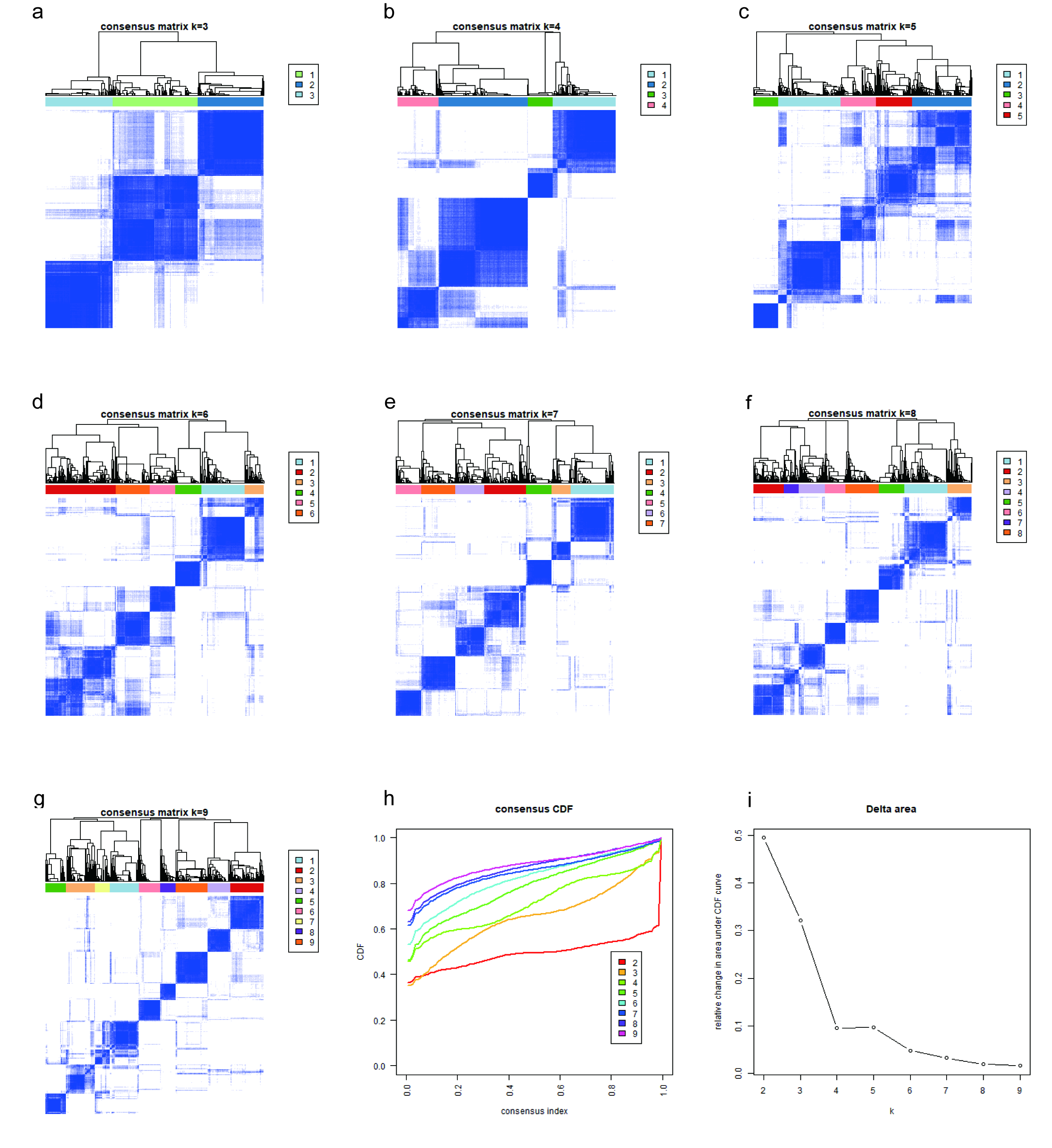

Supplement: Supplementary Figure 2 — Unsupervised clustering of prognostic genes and consensus matrix heat-maps for k = 3-9 through consensus clustering analysis in 732 GC samples. [file Image_2.tif]

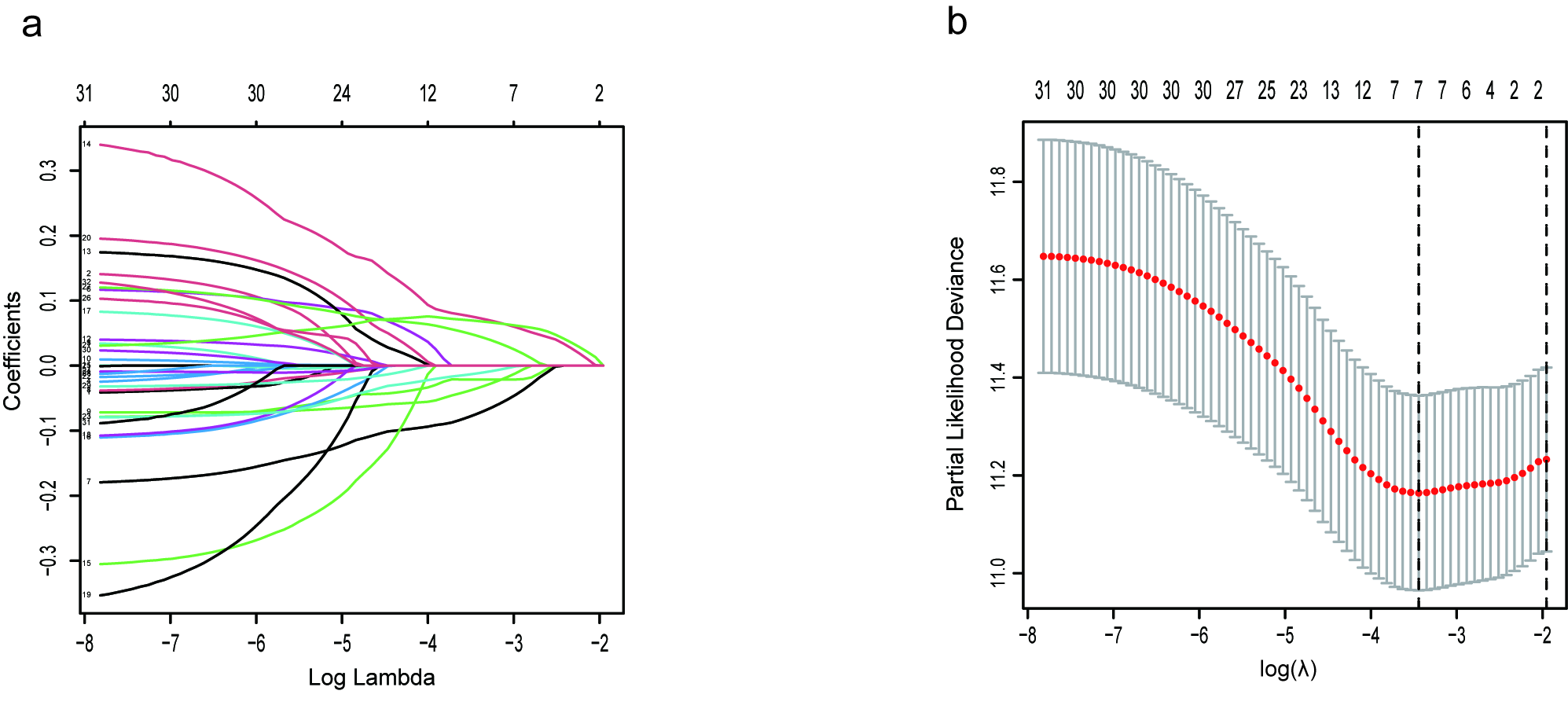

Supplement: Supplementary Figure 3 — Identification of optimum prognostic genes in 732 GC samples. (A, B) The LASSO regression analysis and partial likelihood deviance analysis on 32 subtype-related prognostic DEGs. [file Image_3.tif]

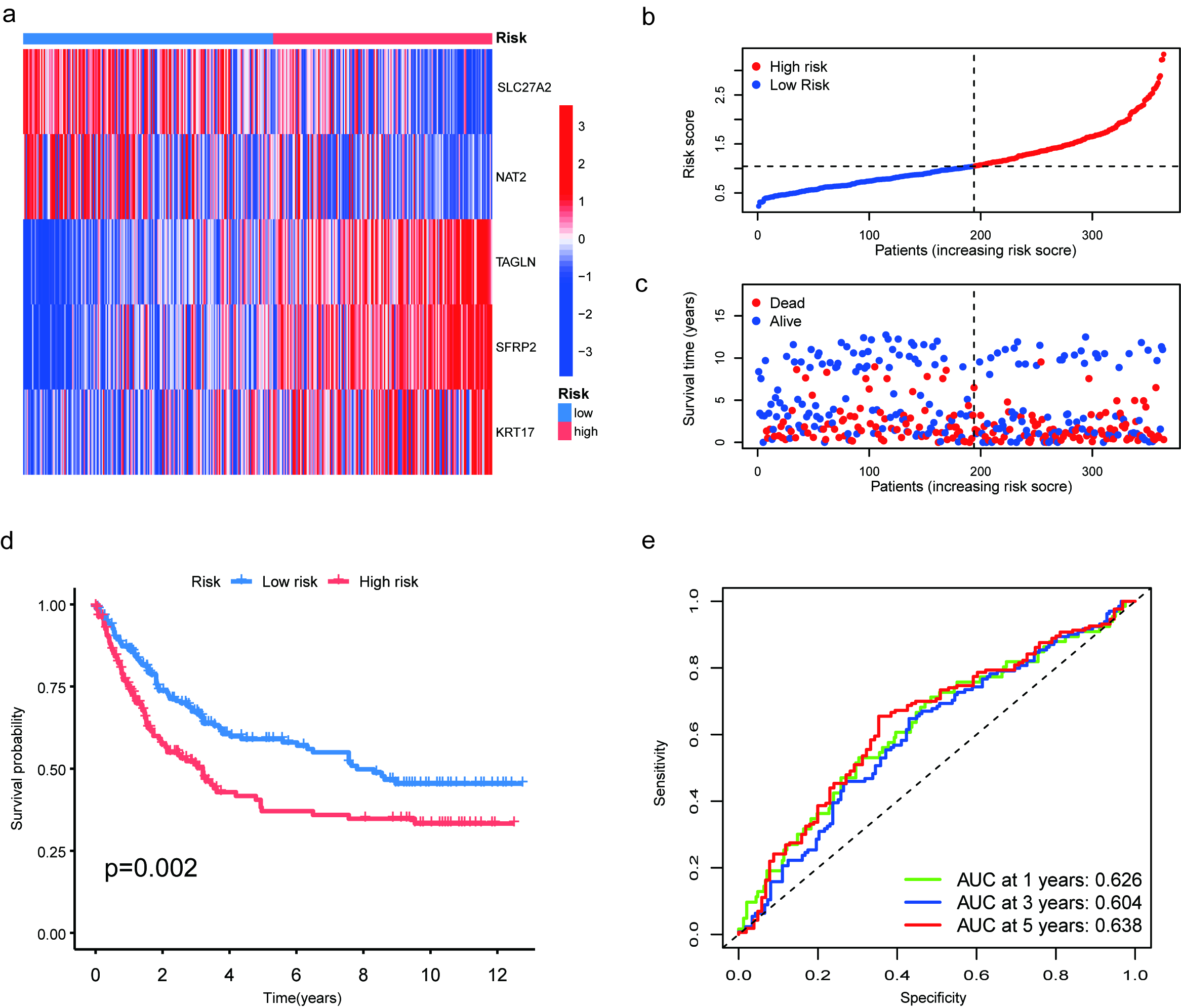

Supplement: Supplementary Figure 4 — Validation of CRG Risk score in the testing group. (A) The heat-map of five scoring genes expression in different risk sets of the testing group. (B, C) Ranked dot and scatter plots of CRG Risk score distribution and patient survival in the testing group. (D) Survival analysis of high- and low- CRG Risk score in the testing group. Kaplan–Meier plot and log-rank tests were conducted for survival analyses. P < 0.05 was considered to be statistically significant. (E) ROC curve predicted the sensitivity and specificity of 1-, 3-, and 5-year survival according to CRG Risk score in the testing group. The testing group included 364 GC samples, among which 170 samples were in high-risk group and 194 samples were in low-risk group. [file Image_4.tif]

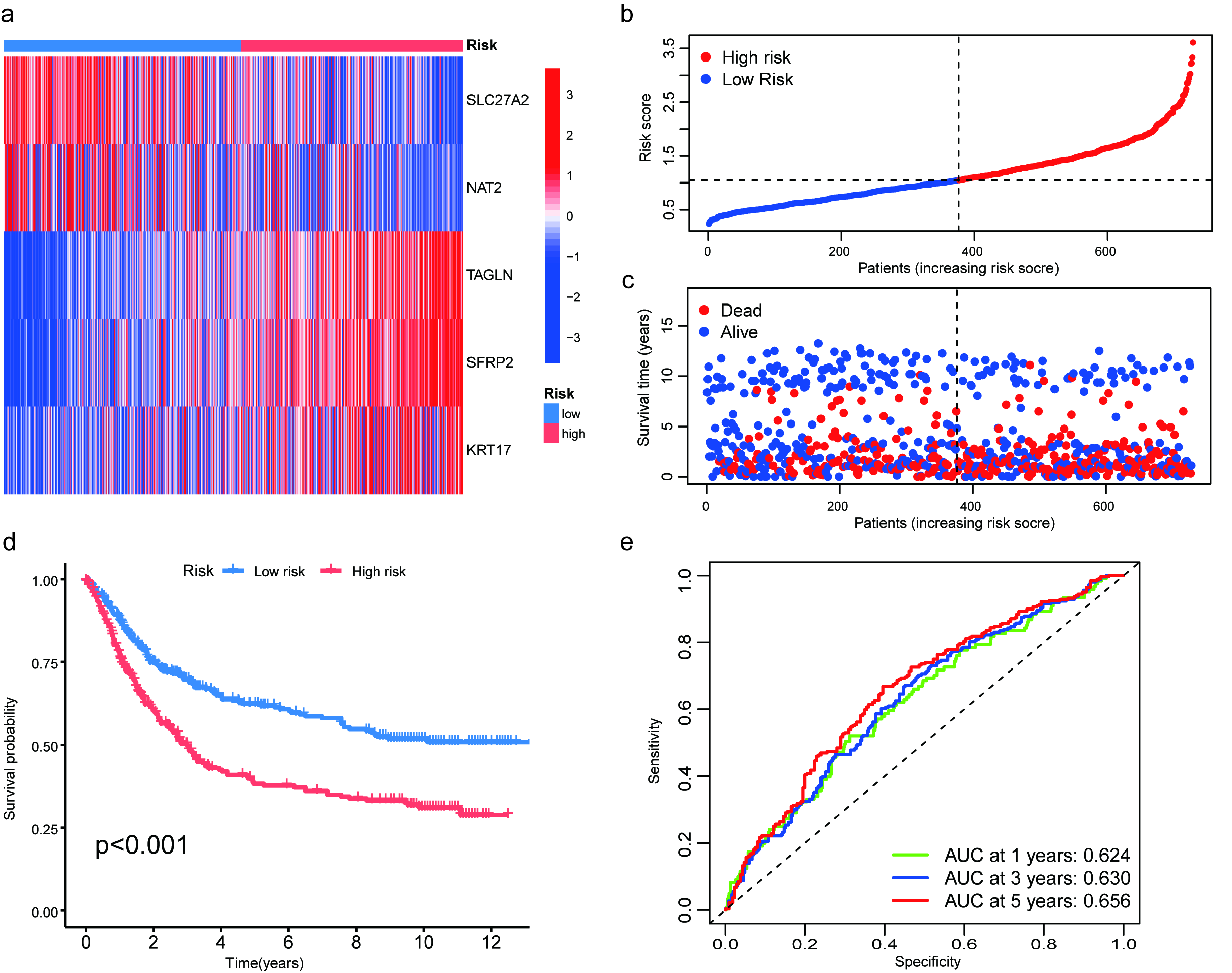

Supplement: Supplementary Figure 5 — Validation of CRG Risk score in the combined TCGA-STAD and GSE84433. (A) The heat-map of the expression profile of five scoring genes in different risk groups. (B, C) Ranked dot and scatter plots of CRG Risk score distribution and patient survival in the combined group. (D) Survival analysis of high- and low- CRG Risk score in the combined group. Kaplan–Meier plot and log-rank tests were conducted for survival analyses. P < 0.05 was considered to be statistically significant. (E) ROC curve predicted the sensitivity and specificity of 1-, 3-, and 5-year survival according to CRG Risk score in the combined group. Combined TCGA-STAD and GSE84433 included 728 GC sample, among which 352 GC samples were in high-risk score group and 376 GC samples were in low-risk score group. [file Image_5.tif]

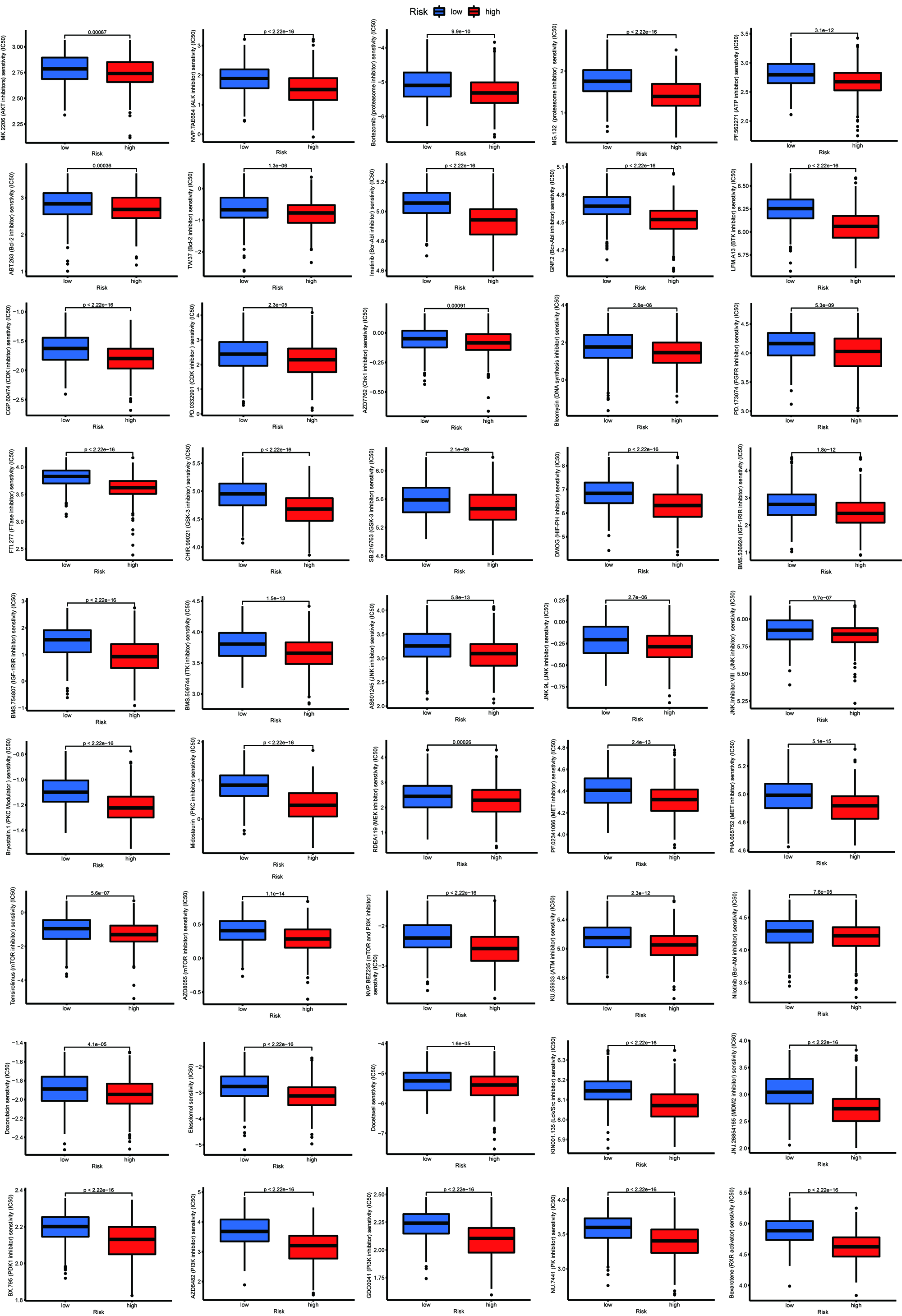

Supplement: Supplementary Figure 6 — Differential drugs susceptibility analyses in high- and low- Risk group. High-risk score group contained 352 GC samples and low-risk score group contained 376 GC samples. P < 0.05 was considered to be statistically significant. [file Image_6.tif]

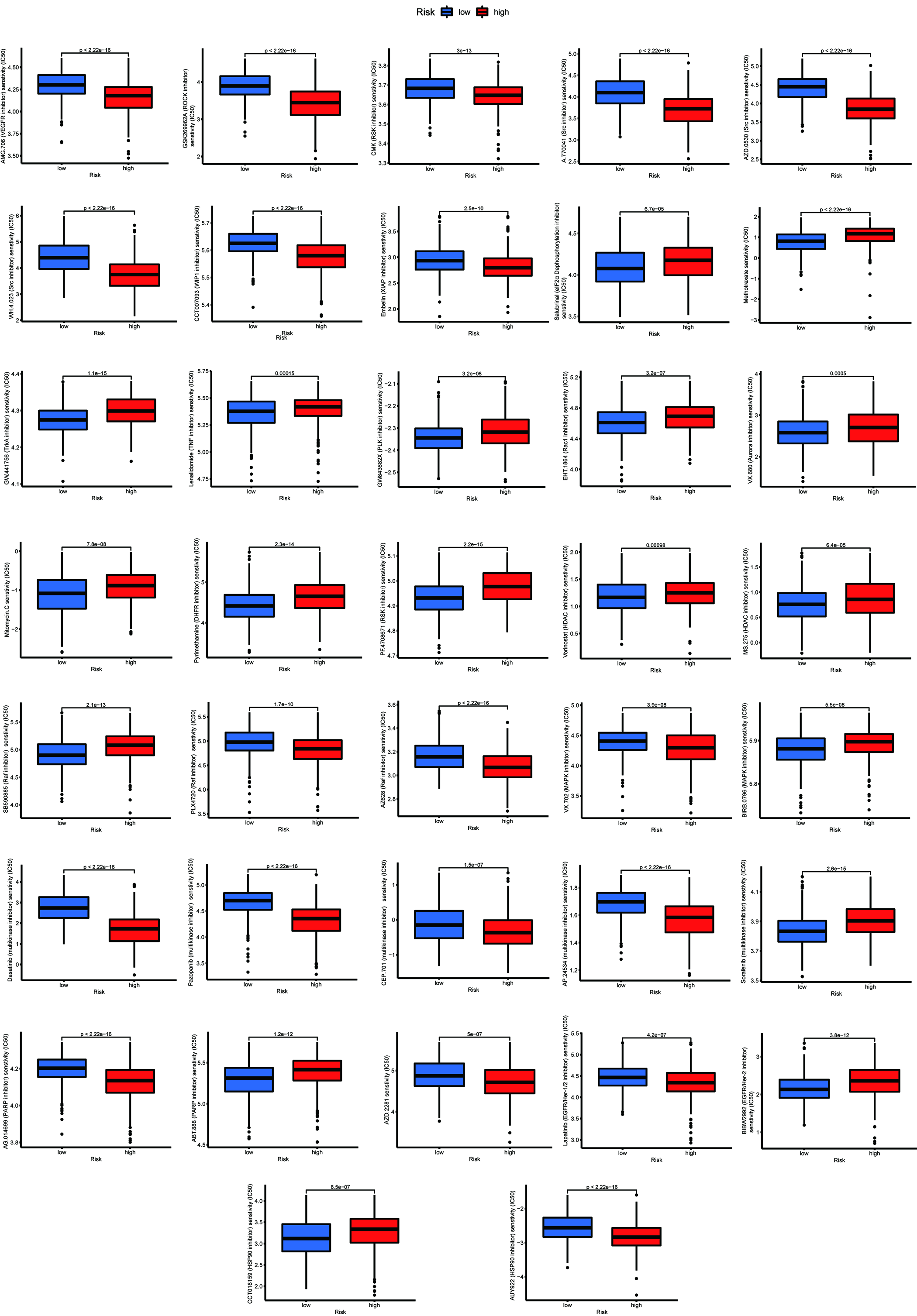

Supplement: Supplementary Figure 7 — Differential drugs susceptibility analyses in high- and low- Risk group. High-risk score group contained 352 GC samples and low-risk score group contained 376 GC samples. P < 0.05 was considered to be statistically significant. [file Image_7.tif]

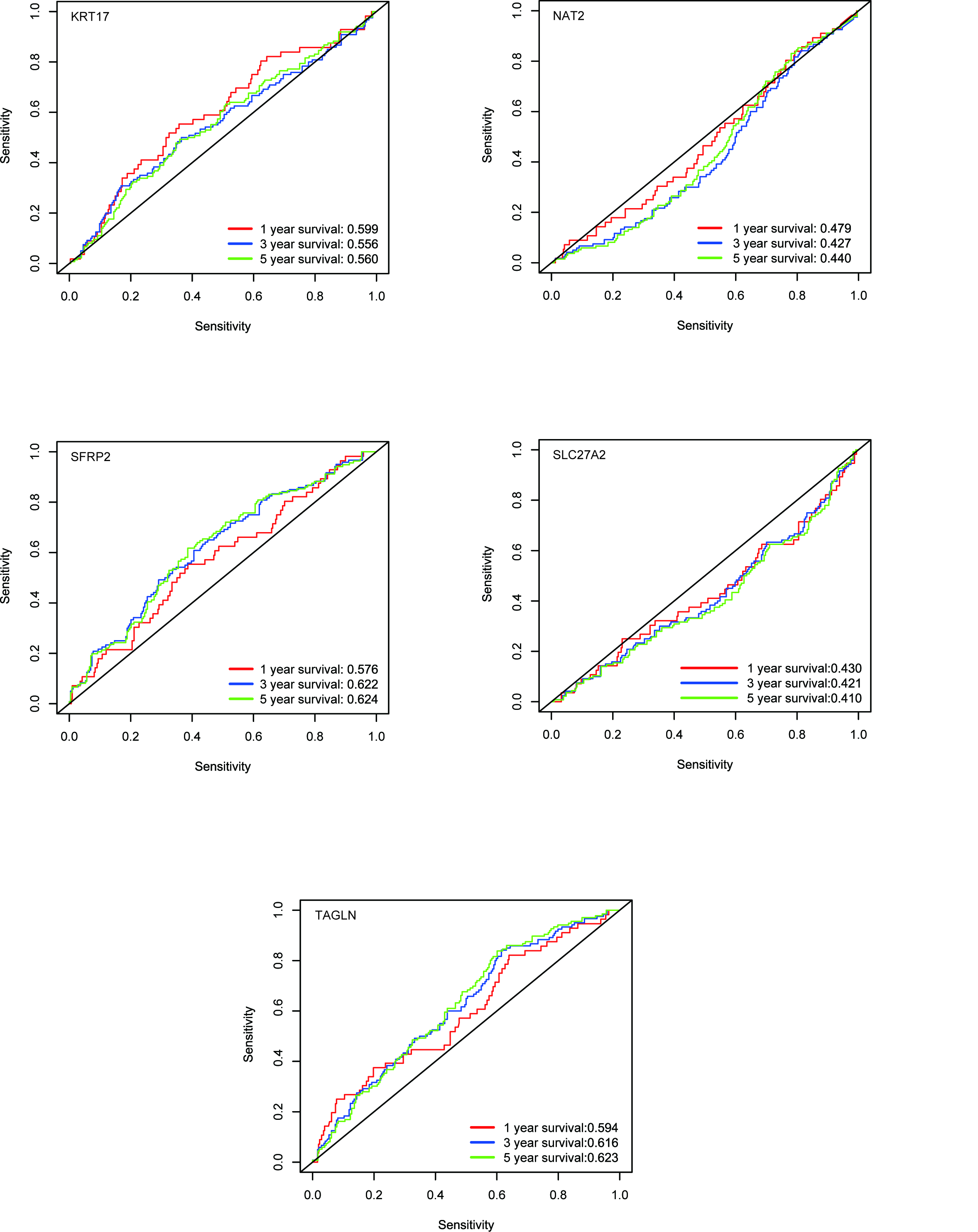

Supplement: Supplementary file 8 [file Image_8.tif]
